# Partners for life

**DOI:** 10.7554/eLife.16798

**Published:** 2016-05-18

**Authors:** Lena Eliasson, Anna Wendt

**Affiliations:** Unit of Islet Cell Exocytosis, Lund University Diabetes Centre, Department of Clinical Sciences in Malmö, Lund University, Malmö, Swedenlena.eliasson@med.lu.se; Unit of Islet Cell Exocytosis, Lund University Diabetes Centre, Department of Clinical Sciences in Malmö, Lund University, Malmö, Sweden

**Keywords:** glucagon, glucagon receptor, insulin, type 1 diabetes, hyperglycemia, glucose homeostasis, Mouse

## Abstract

The hormones insulin and glucagon both play important roles in the development of diabetes.

**Related research article** Damond N, Thorel F, Moyers JS, Charron MJ, Vuguin PM, Powers AC, Herrera PL. 2016. Blockade of glucagon signaling prevents or reverses diabetes onset only if residual β-cells persist. *eLife* **5**:e13828. doi: 10.7554/eLife.13828

**Image** Insulin is produced by β -cells (red) in the pancreas, while glucagon is produced by α-cells (green)

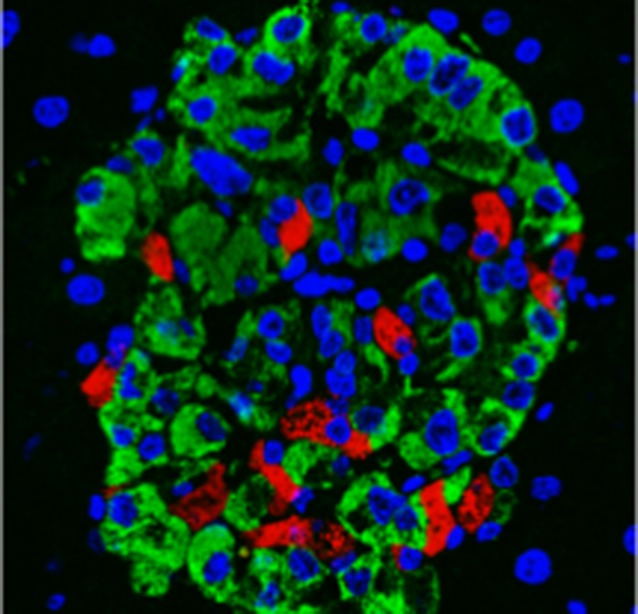

Diabetes is a disease that causes the level of glucose in the blood to become too high. In healthy individuals, two hormones – called insulin and glucagon – work together to keep blood glucose levels within strict limits. Insulin is released when the level of glucose becomes too high, and it stimulates the removal of glucose from the blood so that it can be stored in tissues. On the other hand, glucagon is released when the level of glucose becomes too low, and it triggers the release of glucose from the tissues into the bloodstream.

Many diabetic patients are not able to produce insulin and rely on regular insulin injections to prevent their blood glucose from reaching dangerous levels (insulin-dependent diabetes). Although these injections save lives, they are not sufficient to achieve and maintain the levels of blood glucose that are found in healthy individuals. Even patients considered to have well-controlled diabetes suffer from complications that can damage many tissues in the body. The fact that diabetic patients have too little insulin, as well as uncontrolled levels of glucagon, has led to the hypothesis that diabetes is triggered by inappropriate levels of both hormones, not just insulin alone (Unger and Orci, 1975).

Both insulin and glucagon are produced in the pancreas, within structures called the Islets of Langerhans. Destroying the cells that produce insulin – known as β-cells – causes normal mice to develop diabetes. However, several researchers have recently reported that mice lacking the receptor for glucagon do not develop diabetes when their β-cells are destroyed (Conarello et al., 2007; Lee et al., 2011). These data have attracted a lot of attention since they hold the promise of a new way to treat diabetes, but the conclusions are disputed (Steenberg et al., 2016). Now, in eLife, Pedro Herrera from the University of Geneva and co-workers – including Nicolas Damond as first author – report that inhibiting the action of glucagon to treat diabetes only works if a certain number of β-cells are still present (Damond et al., 2016).

Damond et al. – who are based at the University of Geneva, Eli Lilly, Albert Einstein College of Medicine, Columbia University and Vanderbilt University – made use of mice lacking the glucagon receptor and, in separate experiments, antibodies that can block glucagon signaling. Using these two strategies they were able to elegantly show that if virtually all the β-cells were destroyed, blocking the glucagon signal could not prevent diabetes. However, when the majority, but not all, of the β-cells are destroyed, blocking the glucagon signal could prevent the mice from developing diabetes.

These findings naturally raise the question of whether it is possible to treat diabetes by replacing some of the lost β-cells and administering glucagon inhibitors, instead of giving insulin injections. When faced with severe β-cell loss, the α-cells that normally only produce glucagon can convert to producing both insulin and glucagon (Thorel et al., 2010). Earlier studies show that blocking the glucagon signal increases the number of α-cells (Gelling et al., 2003). Here, Damond et al. show that α-cells are still able to convert to produce both hormones when the glucagon signal is blocked, which results in the Islet of Langerhans having a higher absolute number of α-cells that produce both insulin and glucagon.

How do these findings apply to humans? The experiments make it clear that diabetic patients who cannot produce any insulin would not benefit from a blockade of glucagon signaling. Damond et al. also alert us to the fact that a combination of insulin treatment and blockage of glucagon action might be risky. According to experiments in their laboratories, glucagon signaling is vital to reduce the risk of blood glucose levels becoming too low after insulin injections (unpublished data).

On the other hand, if a patient has enough β-cells to be able to properly respond to changes in blood glucose levels, blocking the glucagon signal might be a useful treatment strategy. Many diabetic patients are not dependent on insulin injections because their β-cells are able to produce some insulin, but not enough to meet the demand. Changes in lifestyle and diet are often effective ways to reduce symptoms in these patients, but it is possible that they could also benefit from receiving drugs that block the glucagon signal.

We know that some patients with insulin-dependent diabetes still have some functional β-cells (Ludvigsson and Heding, 1976). It is not yet clear how many β-cells would be needed for glucagon signal blockers to be an effective alternative to insulin treatment. However, recent advances in understanding how to maintain and/or increase insulin production (Carlsson et al., 2015) provide us with confidence that this milestone will eventually be reached. Although it is not clear what the ideal balance of α-cells and β-cells in the Islets of Langerhans is, the work of Damond et al. tells us that optimal control of blood glucose levels requires these cells to be partners for life.
